# 

**Published:** 1869-07

**Authors:** 


### CONTENTS.

ORIGINAL COMMUNICATIONS.

Thoughts and Experiences on Chloro-Nitrous Oxide for one week.... 273
Contour Fillings—When are they Indicated ?.... 276
Capping Exposed Pulps.... 280
What are the Indications for Extraction ?.... 284

PROCEEDINGS OF SOCIETIES.

Report of Discussions of the Mississippi Valley Dental Association, held March 3, 1869.... 288
The Eleventh Annual Meeting of the Indiana State Dental Association.... 293

SELECTIONS.

Carbolic Acid and its Therapeutical Uses.... 299
Sponge Tents.... 308

CORRESPONDENCE.

.... 309
Chart of the Physiological Arrangement of Cranial Nerves.... 311

EDITORIAL.

Nitrous Oxyd.... 312
Difficulties in Practice.... 315
A Puff.... 316

